# Adherence to screening and referral guidelines for autism spectrum disorder in toddlers in pediatric primary care

**DOI:** 10.1371/journal.pone.0232335

**Published:** 2020-05-07

**Authors:** Kate E. Wallis, Whitney Guthrie, Amanda E. Bennett, Marsha Gerdes, Susan E. Levy, David S. Mandell, Judith S. Miller

**Affiliations:** 1 Division of Developmental and Behavioral Pediatrics, Children’s Hospital of Philadelphia, Philadelphia, PA, United States of America; 2 Perelman School of Medicine, University of Pennsylvania, Philadelphia, PA, United States of America; 3 Center for Autism Research, The Children’s Hospital of Philadelphia, Philadelphia, PA, United States of America; 4 General Pediatrics, The Children’s Hospital of Philadelphia, Philadelphia, PA, United States of America; 5 Center for Mental Health Policy, Perelman School of Medicine, University of Pennsylvania, Philadelphia, PA, United States of America; University of Wyoming College of Health Sciences, UNITED STATES

## Abstract

**Objectives:**

Although the American Academy of Pediatrics recommends screening for autism spectrum disorder (ASD) for all young children, disparities in ASD diagnosis and intervention in minority children persist. One potential contributor to disparities could be whether physicians take different actions after an initial positive screen based on patient demographics. This study estimated factors associated with physicians completing the follow-up interview for the Modified Checklist for Autism in Toddlers with Follow-up (M-CHAT-F), and referring children to diagnostic services, audiology, and Early Intervention (EI) immediately after a positive screen.

**Methods:**

Children seen in a large primary care network that has implemented universal ASD screening were included if they screened positive on the M-CHAT parent questionnaire during a 16–30 month well child visit (N = 2882). Demographics, screening results, and referrals were extracted from the electronic health record.

**Results:**

Children from lower-income families or on public insurance were more likely to have been administered the follow-up interview. Among children who screened positive, 26% were already in EI, 31% were newly referred to EI, 11% were referred each to audiology and for comprehensive ASD evaluation. 40.2% received at least one recommended referral; 3.7% received all recommended referrals. In adjusted multivariable models, male sex, white versus black race, living in an English-speaking household, and having public insurance were associated with new EI referral. Male sex, black versus white race, and lower household income were associated with referral to audiology. Being from an English-speaking family, white versus Asian race, and lower household income were associated with referral for ASD evaluation. A concurrent positive screen for general developmental concerns was associated with each referral.

**Conclusions:**

We found low rates of follow-up interview completion and referral after positive ASD screen, with variations in referral by sex, language, socio-economic status, and race. Understanding pediatrician decision-making about ASD screening is critical to improving care and reducing disparities.

## Introduction

Approximately 1 in 59 children have autism spectrum disorder (ASD) [1]; earlier intervention can lead to improved outcomes [2–4]. To that end, the American Academy of Pediatrics (AAP) guidelines for developmental and ASD screening recommend universal screening for ASD with a standardized tool at 18 and 24-month pediatric visits, or earlier when there are concerns or significant risk for ASD (including having a sibling with ASD) [5]. AAP guidelines recommend that children who screen positive be referred concurrently to early intervention services (EI), audiology, and for comprehensive ASD evaluation [5, 6]. A recent update to AAP guidelines reiterates the importance of early identification and referral for diagnostic evaluation and intervention services [7]. The AAP does not endorse one particular screening tool, but guidelines explicitly state that children who score positive or at risk on the administered tool at any time point should be referred [7].

In the United States, families can get EI services for their children privately and through a federal program for infants and toddlers with disabilities (Part C of the Individuals with Disabilities Education Act, or IDEA), which mandates EI services to qualifying children for a wide range of developmental problems [8]. The EI agency, which operates at the county level, evaluates the child to determine whether they qualify for services. Services can include specialized instruction, speech/language therapy, or physical or occupational therapies, based on either the child’s developmental delays or on known developmental risks associated with specific diagnoses. Children qualify for EI services when they show any developmental delay (e.g., motor or language delays), which often is apparent before an ASD diagnosis is confirmed [5]. Once an ASD diagnosis is confirmed, the intervention team may add ASD-specific services such as behavioral therapy.

While rates of developmental and autism-specific screening have increased in the US, disparities in the proportion and ages of diagnosis of minority children with ASD persist [9, 10]. For example, the CDC recently reported that white children were 22% more likely to be identified with ASD than were Hispanic children [1]. When minority children with autism are identified, they tend to be more severely impaired [11–13], suggesting that those less severely impaired are missed altogether. Among those who are diagnosed, minority children tend to be identified later than white children [14]. In a promising development, recent CDC surveillance data suggests some progress, as they did not find racial or ethnic differences in median age of diagnosis [1], or differences in prevalence rates between non-Hispanic black and white children [15].

Gender is another area of potential disparity, with girls with ASD less likely than boys to be diagnosed [16]. Although ASD is more common in boys than in girls [17], differences in the phenotype by gender suggest that girls may be under-identified and that girls with average to above-average intelligence may be under-represented among children diagnosed with ASD [16, 18].

Two recent studies have examined referrals after ASD screening. Monteiro et al., found that after a positive ASD screen, only 31% of children were referred for a diagnostic evaluation, 20% to EI, and 36% to audiology [19]. They did not find an association between race, ethnicity, or insurance status on referrals for ASD evaluation [19], but did not report on socio-demographic predictors of referral to EI or audiology. Rea et al. [20] found inconsistencies in referrals after positive ASD screen, but did not find ethnic disparities in who was referred. In the present study, we examine a similar question, more closely examining the possibility of disparities in a diverse patient population cared for in urban, suburban and rural sites across a large primary care network. Prior studies of referrals after developmental screening suggest that patient characteristics such as race, ethnicity, or gender may influence physician response. For example, one vignette-based study found that a girl with language delay was 60% more likely to be referred to audiology than a boy with the same presenting symptoms [21]. Among a sample of very-low-birthweight infants eligible for EI, referrals were significantly lower among the children of black mothers, mothers without private insurance, or from towns with higher poverty rates [22].

The Children’s Hospital of Philadelphia (CHOP) has a well-implemented universal developmental and ASD-specific screening program. Among a cohort of nearly 26,000 children seen for well-child care between age 16–26 months, 91% were screened with the Modified CHecklist for Autism in Toddlers with the follow-up interview available (M-CHAT-F) [23]. CHOP’s program includes electronic access to the M-CHAT follow-up interview, unlike the programs described in the Monteiro et al., and Rea et al., studies, which did not use the follow-up interview in clinical practice. While our overall screening rate is very high, the subgroup of children who did not complete screening were more likely to be non-white, have public insurance, be from lower-income households, or be from homes where languages other than English are spoken [23].

The present study builds on our prior work evaluating the completion and accuracy of the M-CHAT-F across the CHOP Primary Care Network. In the present study, we examine a cohort of children with positive screens to estimate rates of completion of the M-CHAT follow-up interview and adherence to AAP guidelines to refer to EI, audiology, and for an ASD evaluation immediately after a positive M-CHAT-F screen. We also examined the potential contribution of general developmental screening results (described below) alongside ASD screening results. We hypothesized that patient socio-demographic characteristics such as racial, ethnic, socio-economic, gender, and language-based differences would affect the likelihood of physicians completing the follow-up interview and referring children after a positive M-CHAT-F screen.

## Methods

CHOP’s primary care network includes 31 sites serving a geographically, socioeconomically and racially diverse population in Pennsylvania and New Jersey. CHOP established universal developmental and autism-specific screening across its primary care network in 2011, through electronic administration of parent questionnaires and clinical decision support built directly in the electronic health record (EHR). In 2013, CHOP added the *Survey of Well-being of Young Children (SWYC)* Milestones as a general development screen. The AAP recommends both ASD-specific and general developmental screening to capture different aspects of a child’s development [7].

The SWYC Milestones questionnaires include 10 age-specific items to evaluate children’s attainment of motor, cognitive, and language skills. The appropriate questionnaires for 15, 18, 24, and 30-month ages were assigned according to published age rules. For each item, parents respond “not yet,” “somewhat,” or “very much.” Scores are compared to age-based norms, with higher scores indicating attainment of additional skills. Sensitivity for the SWYC milestones at 18–30 months ranges from 67 to 81%, and specificity ranges from 71 to 88% for each age-normed questionnaire [24].

The M-CHAT-F is a 23-item parent-report tool that asks yes/no questions about a child’s behaviors to determine risk of ASD [25]. The follow-up interview items reduce the false-positive rate, and are indicated when scores are in the moderate-risk range (scores of 3–7). For scores of 8 or higher, the follow-up interview is not required, because these higher scores signal likelihood of a developmental challenge that needs additional diagnostic evaluation. The updated M-CHAT-R/F has slightly different wording and scoring, but very comparable accuracy, such that data between the M-CHAT-F and M-CHAT-R/F are often combined [26]. Thus, CHOP elected to continue using the M-CHAT-F rather than update to the M-CHAT-R/F.

As part of routine care, caregivers completed SWYC and M-CHAT-F questionnaires on an electronic tablet or through an electronic patient portal prior to designated well-child visits. Results automatically populated the EHR visit report, including a link through which the provider could access the relevant M-CHAT follow-up interview items for completion during the visit. Physicians could choose not to administer the follow-up interview.

**Fig 1:**
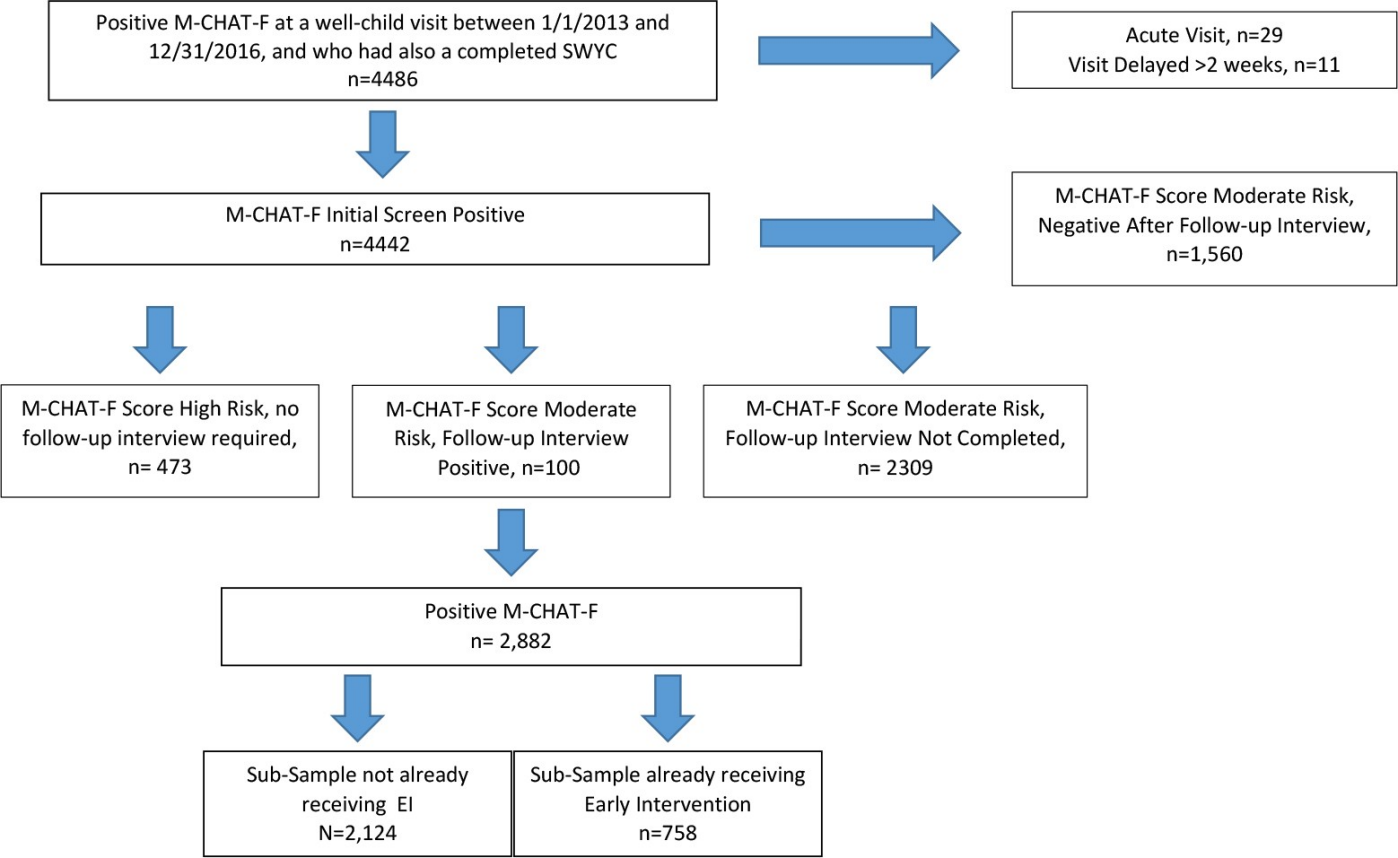
Sample inclusion for final analysis.

We identified all children aged 16–30 months who screened positive on the M-CHAT-F between January 1, 2013 and December 31, 2016 and who also had received the SWYC (n = 4486, *see Fig 1*). We selected this cohort of children with both M-CHAT-F and SWYC results so that we could include SWYC scores as a co-variate in analyses as a measure of development and to ensure that physicians had access to the same information about the developmental status of all children when making referral decisions. Children were excluded if the screening was completed at an acute care visit (n = 29), or if the visit itself was delayed more than 2 weeks after M-CHAT-F was completed online (n = 11).

The M-CHAT-F was considered positive if the total score was 3 or more, or if 2 of the 6 original “critical items” were endorsed. For children who had multiple visits in the EHR that met these criteria, only the visit with the first positive M-CHAT-F was included, as actions at this visit reflect earliest opportunity to accelerate diagnosis and intervention.

Our final sample of 2882 children included all children whose visit ended with a documented positive screen, regardless of whether the provider used the follow-up interview to come to that conclusion (see below). We could identify which children had previously been referred or were already receiving EI services (n = 758; 26.3%), but not which children had already received a referral for audiology or an ASD evaluation. Thus, analyses reflect subgroups where relevant. The Institutional Review Board at The Children's Hospital of Philadelphia reviewed and approved the research, including a waiver of consent because our study comprised retrospective analysis of EHR data.

A trained data analyst extracted the following data directly from the EHR: M-CHAT-F scores (continuous); SWYC results (screen positive/negative); date of visit; age of child at visit; patient address at the time of M-CHAT-F visit; parent-reported race, ethnicity, and languages (up to three); site of primary care visit; and insurance payer (public versus private). Data from the US Census Bureau linked patient addresses at the census tract-level to statistics on median household income; data were accessed at https://data.census.gov with inflation-adjusted terms for the year in which the M-CHAT-F was administered.

Other than the existence of an earlier EI referral, immediate actions–defined as occurring during the visit where screening occurred—were the focus of our study. Referrals to EI reflect referrals to public agencies that provide federally-mandated services. Thus, we electronically extracted the following from the screening visit: orders placed for EI or other specialists, referral letter written for EI, and provider write-in response to an EHR-prompted question about actions taken in response to positive M-CHAT-F or mentioned in the after-visit summary. We categorized written responses as follows. A referral documented in any of the above locations in the EHR was considered “referred.” When documentation suggested that parents refused referral to EI, those patients were still included in the “referred to EI” category because a referral was attempted. We considered children to have been referred for ASD/additional evaluation if a referral to any of the following specialties was documented, within or outside of the CHOP Network: speech/language therapy, occupational therapy, developmental-behavioral pediatrics, neurology, psychiatry, psychology, or genetics. We included a broad range of specialists to be as inclusive as possible for those who might be able to confer a clinical ASD or additional relevant diagnosis in the network, and because these are the specialists to whom our primary care providers might choose to send patients for evaluation. Although the new ASD-screening guidelines recommend referral to audiology only when there are concerns about attention or language [7], the prior screening algorithm recommended referral for all children with a positive ASD screen [5], so we evaluated adherence to the guideline in place at the time of the positive screen.

One author (KEW) randomly selected 10% of charts to review manually to confirm data accuracy, and to determine whether we should consider data from any other location in the EHR. We manually extracted M-CHAT-F and SWYC scores. There was >90% agreement between manually extracted and digitally extracted referral data. Scores on the M-CHAT-F and SWYC showed 100% agreement for manually and digitally extracted data.

Respondent characteristics (socio-demographics) were a mix of categorical and continuous variables (*see Table 1*). Race was categorized as white, black, Asian, and “other” (other races, including multiple reported races, were combined because of small sample sizes; 11 patients with unknown race were included in “other”). A binary variable for language exposure was created for “English-only” versus “multiple languages or non-English.” Median household income at the census-tract level was categorized by quartiles.

**Table 1:** Sample characteristics (N = 4442).

Categorical Variables: Number (percent)	
Sex:	
Male	2453 (55.2%)
Female	1989 (44.8%)
Race:	
White	1140 (25.7%)
Black/African-American	1969 (44.3%)
Asian	385 (8.7%)
Other	948 (21.3%)
Ethnicity:	
Hispanic	541 (12.2%)
Non-Hispanic	3893 (87.6%)
Missing	8 (0.2%)
Language spoken at home:	
English-only	3866 (87.0%)
Non-English/Multiple	558 (12.6%)
Missing	18 (0.4%)
Insurance Payor:	
Private	1430 (32.2%)
Public/Medicaid	2955 (66.5%)
Missing	57 (1.2%)
Provider Type:	
Attending Physician	3453 (77.7%)
Nurse Practitioner	605 (13.6%)
Resident	298 (6.7%)
Missing	86 (1.9%)
Survey of Wellbeing in Young Children (SWYC) Results:	
SWYC Negative	2465 (55.5%)
SWYC Positive	1977 (44.5%)
Continuous Variables: Median (Interquartile Range, IQR)^a^	
Patient Age at time of M-CHAT-F^a^ administration in months	19 (18–23)
Median Household Income (Census-Tract Level)	$47518 ($31001–71379)
Modified CHecklist for Autism in Toddlers (M-CHAT-F) Score	4 (3–5)

^a^ Data are not normally distributed

Logistic regression, which included practice site as a fixed effect, was used to compare 1) characteristics of children who received or did not receive the follow-up interview, and 2) those who screened positive or negative after the follow-up interview when administered. A Bonferroni correction was used to account for multiple comparisons, with p < .006 considered statistically significant.

Chi-square analyses were used to compare referrals among children who had previously initiated EI and those who had not. Among children previously receiving EI, children were considered to have received all recommended referrals if they were newly referred to audiology and for additional ASD evaluation. Among children not previously receiving EI, children were considered to have received all recommended referrals if they were referred to EI, audiology and for additional ASD evaluation.

Variables were included in multivariable logistic regression models based on a priori hypotheses using a nested design, to account for patient clustering within primary care practice sites. Variables were removed to improve model fit. Multivariable logistic regression was used to compare characteristics associated with referrals to audiology and for comprehensive ASD evaluation. Models were adjusted for time from study initiation as a fixed effect to account for changes in practice over time. Multinomial, multivariable logistic regressions were used to compare three groups: 1) children who were documented to have already been referred to EI or were already receiving EI services at the time of the M-CHAT-F administration; 2) children who were referred to EI on the day of the positive M-CHAT-F; 3) children who were not referred to EI prior to or on the day of the screening visit. These models adjusted for county of family residence and a measure of time, to account for differences in EI referral practice based on county EI agency and changes in practice over time, respectively, as fixed effects. Tests of significance for multivariable models were 2-tailed with alpha set to .05. All data were analyzed in Stata, version 15 [27].

## Results

### Follow-up interview completion

Children with high-risk scores (n = 473, 10.6% of 4442 with positive scores on M-CHAT-F initial screen) did not require the follow-up interview, because their high score signals a need for additional diagnostic evaluation. An additional 3969 children had moderate-risk scores. Of these, clinicians completed the M-CHAT-F follow-up interview for 1660 (41.8% of 3969, *see Table 2*). Factors associated with increased likelihood of follow-up interview completion included: public versus private insurance (p < .001), being seen by a resident or nurse practitioner versus attending physician (p < .001), having a concurrently positive developmental screen (p < .001), and being from a family with lower median household income (estimated at the neighborhood level) (p < .001).

**Table 2:** Regression analysis: Socio-demographic factors associated with completion of follow-up interview (n = 3969), positive follow-up interview (n = 1660).

	Percent Who Completed Follow-Up Interview	p value ^a^	For those who completed follow-up interview, follow-up interview positive	p value ^a^
Number (percent)	1660 (41.8%)	-	100 (6.0%)	-
Sex:				
Male	42.1%	0.75	67 (7.4%)	0.01
Female	41.5%		33 (4.4%)	
Race:				
White	32.1%		24 (7.6%)	
Black/African-American	51.4%	0.5	54 (5.9%)	0.13
Asian	36.7%		6 (4.6%)	
Other	35.3%		14 (4.7%)	
Ethnicity:				
Hispanic	37.0%	0.10	9 (5.1%)	0.60
Non-Hispanic	42.5%		91 (6.2%)	
Language spoken at home:				
English-only	41.9%	0.79	92 (6.4%)	0.14
Non-English/Multiple Languages	41.6%		8 (3.7%)	
Insurance Payor:				
Private	33.2%	<0.001	27 (6.4%)	0.57
Public/Medicaid	46.3%		69 (5.7%)	
Provider Type:				
Attending Physician	38.8%		77 (6.5%)	0.68
Nurse Practitioner	45.9%	<0.001	13 (5.1%)	
Resident	65.5%		8 (4.6%)	
SWYC^b^ Results:				
SWYC Negative	29.8%	<0.001	31 (2.6%)	<0.001
SWYC Positive	49.5%		69 (15%)	
Continuous Variables (Median, Interquartile Ranges for data not normally distributed)				
Patient Age at time of M-CHAT-F^c^ administration in months	Completed Follow-Up Interview: 19 (18–24)		Negative: 19 (18–24)	
	Did not Complete Follow-Up Interview: 18 (18–23)	0.99	Positive: 18 (18–20)	0.004
Median Household Income (Census-Tract Level)	Completed Follow-Up Interview: $37,875 ($28,201–61,868)		Negative: $37,411 ($28,201–61,304)	
	Did not Complete Follow-Up Interview: $53,298 ($33,004–77,135)	<0.001	Positive: $41,595 ($27,310–69,114)	0.54

^a^ Regression analysis of association between completion of demographic variable and completion of follow-up interview, adjusted by practice site. Tests of association were considered significant at the p = .006 level (Bonferroni correction to account for multiple comparisons).

^b^ Survey of Wellbeing in Young Children (SWYC)

^c^ Modified CHecklist for Autism in Toddlers (M-CHAT-F)

### Follow-up interview positive

Among those who completed the follow-up interview, 100 (6.0%) children continued to screen positive. Having a concurrently positive SWYC (p < .001) and being younger at time of screen (p = .004) were associated with higher likelihood of screening positive on the follow-up interview. For children for whom the follow-up interview was administered and was positive (n = 100), 66% of children with a positive follow-up interview were referred to EI, 48% were referred for additional ASD evaluation, and 40% were referred to audiology.

### Referral rates

Among the 2882 children who screened positive on the M-CHAT-F, 26.3% (n = 758/2882) were already receiving EI, and another 31.0% (894/2882) were newly referred to EI, for a total of 57.3% (n = 1652/2882); 10.7% (n = 308/2882) were referred to audiology, and 11.4% (n = 328/2882) were referred for additional evaluation; categories are not mutually exclusive. Among those not already receiving EI, 44.8% (n = 951/2124) received at least 1 referral the day of the positive screen, but only 3.2% (n = 67) received all 3 recommended referrals; 10.3% (n = 219/2124) were referred to audiology and 8.8% (n = 187/2124) were referred for additional evaluation. Among children previously referred to EI, 5.1% (n = 39/758) received the other 2 recommended referrals: 11.7% (n = 89/758) to audiology and 18.1% (n = 137/758) for additional evaluation. Across the entire sample, only 3.7% (106/2882) left the visit with all three recommended referrals. *(See Table 3 for statistical comparisons.)*

**Table 3:** Referrals made on day of visit for children with positive ASD screen.

Referral^a^	Number (Percent) Referred- Not Already Receiving EI (n = 2124)	Number (Percent) Referred- Already Receiving EI (n = 758)	Number (Percent) of Total Sample Referred (n = 2882)	P value^b^
Referred to Early Intervention (EI) in Visit	894 (42.1%)	N/A	N/A	N/A
Referred to Audiology in Visit	219 (10.3%)	89 (11.7%)	308 (10.7%)	0.3
Referred for ASD evaluation^c^	187 (8.8%)	137 (18.1%)	324 (11.2%)	<0.001
Received at least 1 referral	951 (44.8%)	208 (27.4%)	1159 (40.2%)	<0.001
Received all recommended referrals	67 (3.2%) (Early Intervention, audiology and comprehensive ASD evaluation)	39 (5.1%) (Audiology and comprehensive ASD evaluation only)	106 (3.7%)	0.01

^a^ Categories are not mutually exclusive

^b^ Chi-Square comparison for those Already Receiving EI and those Not Already Receiving EI

^c^ Examples of additional referrals include: Speech/language therapy, occupational therapy, developmental-behavioral pediatrics, neurology, psychiatry, psychology, or genetics.

In adjusted analyses *(see Table 4),* the relative risk of having been referred to EI prior to the screening visit was higher for children with concurrently positive general developmental screen (adjusted relative risk ratio, [aRRR] = 26.89, 95% Confidence Intervals, [CI] 19.89–36.35, p < .001), children exposed to only English at home versus non-English or multiple languages (aRRR = 2.18, 95% CI = 1.38–3.44, p < .001), for children who were older at the time of screening (aRRR = 1.05, 95% CI = 1.01–1.09, p = .02), and for children with higher M-CHAT-F score (aRRR = 1.29, 95% CI = 1.22–1.35, p < .001). The adjusted relative risk of having previously been referred to EI prior to the screening visit was lower for black (aRRR = 0.53, 95% CI = 0.37–0.76, p < .001), Asian (aRRR = 0.31, 95% CI = 0.19–0.52, p < .001), and “other” races (aRRR = 0.55, 95% CI = 0.38–0.80, p = .002) versus white race. Other socio-demographic factors were not statistically significantly associated with prior EI initiation in adjusted models.

**Table 4:** Referral status to early intervention (EI) after positive M-CHAT-F by demographics (n = 2,882)^a^.

	Number (percent) Not Referred to Early Intervention prior to or during visit	Number (percent) Referred to Early Intervention Prior to Visit	Adjusted Relative Risk Ratio^b^	95% Confidence Interval	p value	Number (percent) Referred to Early Intervention in Visit	Adjusted Relative Risk Ratio^b^	95% Confidence Interval	p value
Total Sample	1230 (42.7%)	758 (26.3%)	-	-	-	894 (31%)	-	-	-
Race:									
-White	285 (33.6%)	309 (36.4%)	Reference	Reference	Ref	255 (30.0%)	Reference	Reference	Ref
-Black/African-American	491 (44.2%)	266 (24.0%)	0.53	0.37–0.76	<0.001	353 (31.8%)	0.67	0.48–0.93	0.02
-Asian	145 (55.8%)	35 (13.5%)	0.31	0.19–0.52	<0.001	80 (30.8%)	0.73	0.48–1.09	0.12
-Other	309 (46.6%)	148 (22.3%)	0.55	0.38–0.80	0.002	206 (31.1%)	0.84	0.60–1.16	0.29
Ethnicity:									
-Hispanic	158 (42.5%)	101 (27.2%)	1.41	0.91–2.18	0.12	113 (30.4%)	1.01	0.69–1.48	0.96
-Non-Hispanic	1069 (42.7%)	656 (26.2%)	Reference	Reference		780 (31.1%)	Reference	Reference	
Sex:									
-Male	591 (36.7%)	452 (28.1%)	1.26	0.99–1.61	0.07	566 (35.2%)	1.6	1.30–1.98	<0.001
-Female	639 (50.2%)	306 (24.0%)	Reference	Reference		328 (25.8%)	Reference	Reference	
Language:									
-English-only	1041 (41.4%)	691 (27.5%)	2.18	1.38–3.44	<0.001	785 (31.2%)	1.65	1.14–2.40	0.008
-Non-English/Multiple	180 (51.1%)	66 (18.8%)	Reference	Reference		106 (30.1%)	Reference	Reference	
Insurance:									
-Private	440 (42.6%)	309 (29.9%)	Reference	Reference	Ref	284 (27.5%)	Reference	Reference	Ref
-Public/Medicaid	771 (42.7%)	441 (24.5%)	0.95	0.71–1.28	0.75	592 (32.8%)	1.35	1.04–1.75	0.02
Provider Type:									
-Attending Physician	987 (42.2%)	644 (27.5%)	N/A^c^	N/A^c^	N/A^c^	709 (30.3%)	N/A^c^	N/A^c^	N/A^c^
-Nurse Practitioner	174 (47.9%)	71 (19.6%)				118 (32.5%)			
-Resident	46 (35.1%)	35 (26.7%)				50 (38.2%)			
SWYC^d^ Results:									
-Positive SWYC	234 (14.8%)	688 (43.4%)	26.89	19.89–36.35	<0.001	664 (41.9%)	10.9	8.72–13.64	<0.001
-Negative SWYC	996 (76.9%)	80 (5.4%)	Reference	Reference		230 (17.8%)	Reference	Reference	
Median Household Income (Census-Tract Level) quartiles									
0-25^th^ %ile			1.04	0.66–1.64	0.88		1.31	0.88–1.96	0.19
25^th^-50^th^ %ile			0.99	0.66–1.47	0.95		1.24	1.87–1.75	0.24
50-75^th^ %ile			1.03	0.73–1.45	0.89		1.27	0.93–1.73	0.13
75-100^th^ %ile			Reference	Reference	Ref		Reference	Reference	Ref
Continuous Variables (Median, Interquartile Ranges for data not normally distributed)									
M-CHAT-F^e^ Score	3 (3–4)	6 (4–9)	1.29	1.22–1.35	<0.001	4 (3–6)	1.13	1.07–1.19	<0.001
Median Household Income (Census-Tract Level)	$51,797 ($32737–75,655)	$57,085 ($34,495–78,500)	N/A^f^	N/A^f^	N/A^f^	$50,096 ($31,572–72,584)	N/A^f^	N/A^f^	N/A^f^
Age in Months	19 (18–23)	19 (18–24)	1.05	1.01–1.09	0.02	19 (18–23)	0.98	0.94–1.01	0.23

^a^All models adjusted for county and time from study initiation

^b^Reference category in multinomial multivariable logistic regression is group not referred to early intervention prior to or during visit

^c^Variable not included in multivariable models, as inclusion led to poorer model fit

^d^Survey of Wellbeing in Young Children (SWYC)

^e^Modified CHecklist for Autism in Toddlers (M-CHAT-F)

^f^Quartile data for income included in models, as continuous data led to poorer model fit given distribution of sample

The odds of being referred to EI or audiology during the visit were higher for boys than for girls (for EI: adjusted odds ratio [aOR] = 1.60, 95% CI = 1.30–1.98, p < .001; and for audiology: aOR = 1 .50, 95% CI 1.15–1.95, p = .003); but there was no statistically significant difference in referral for additional ASD evaluation based on sex (*see Tables 4 and 5)*. The odds of referral to EI, audiology or ASD evaluation were higher for children who also screened positive on the SWYC milestones (for EI: aOR = 10.90, 95% CI 8.72–13.64 *p* < .001; for audiology: aOR = 4.45, 95% CI 3.21–6.17, p < .001; for ASD evaluation: aOR = 4.85, 95% CI 3.39–6.92, p < .001). The odds of referral to EI and for additional ASD evaluation were higher for children who had English-only documented in the EHR (for EI: aOR = 1.65, 95% CI 1.14–2.40, p = .008; for ASD evaluation: aOR = 2.21, 95% CI 1.23–3.97, p = .01); there was no statistically significant difference in referral for audiology based on language. Children with higher M-CHAT-F scores were more likely to be referred to EI (aOR = 1.13, 95% CI 1.07–1.19, p < .001) or for ASD evaluation (aOR = 1.10, 95% CI 1.07–1.15, p < .001). Black children were less likely than white children to be referred to EI (aOR = 0.67, 95% CI 0.48–0.93, p *=* .02), but were more likely than white children to be referred to audiology (aOR = 1.50, 95% CI 1.03–2.19, p = .04). Asian children were less likely to be referred for additional ASD evaluation (aOR = 0.43, 95% CI 0.21–0.86, p = .02). Other races and ethnicities did not statistically differ in likelihood of each referral. Compared with children with median household income (estimated at the neighborhood level) in the highest quartile, children from the lowest income households were more likely to be referred for audiology and ASD evaluation (lowest quartile median household income versus highest quartile for audiology: aOR = 3.10, 95% CI 1.92–5.00, p < .001; for ASD evaluation: aOR = 1.57, 95% CI 1.01–2.44, p = .045). Children with public insurance were more likely to be referred to EI (aOR = 1.35, 95% CI 1.04–1.75, p = .02). Child age and Hispanic ethnicity were not statistically significantly associated with any referral in adjusted analyses. Interactions between race and SWYC score, and race and household income, were not statistically significant.

**Table 5:** Multivariable regression analysis: Factors related to likelihood of referral to audiology and for ASD evaluation after positive M-CHAT-F, (n = 2882)^a^.

Multivariable Logistic Regression Analysis: Nested by Practice Site						
Dependent Variable: Referral to Audiology and/or ASD evaluation during visit with positive M-CHAT-F						
	Referral to Audiology			Referral for ASD Evaluation		
	n = 308 (10.7%)			n = 324 (11.2%)		
	Adjusted Odds Ratio	95% Confidence Intervals	p value	Adjusted Odds Ratio	95% Confidence Intervals	p value
Race:						
-White	Reference	Reference	Ref	Reference	Reference	Ref
-Black/African-American	1.50	1.03–2.19	0.04	1.07	0.75–1.52	0.73
-Asian	1.33	0.76–2.31	0.32	0.43	0.21–0.86	0.02
-Other	0.75	0.47–1.19	0.22	0.82	0.55–1.22	0.32
Ethnicity:						
-Hispanic	1.14	0.71–1.83	0.58	1.138	0.76–1.84	0.47
-Non-Hispanic	Reference	Reference	Ref	Reference	Reference	Ref
Sex:						
-Male	1.50	1.15–1.95	0.003	1.27	0.98–1.64	0.07
-Female	Reference	Reference	Ref	Reference	Reference	
Language:						
-English-only	1.24	0.74–2.08	0.42	2.21	1.23–3.97	0.01
-Non-English/Multiple	Reference	Reference	Ref	Reference	Reference	Ref
Insurance:						
-Private	0.74	0.53–1.03	0.07	1.00	0.73–1.36	0.98
-Public/Medicaid	Reference	Reference	Ref	Reference	Reference	Ref
Median Household Income (Census-Tract Level) quartiles:						
0-25^th^ %ile	3.10	1.92–5.00	<0.001	1.57	1.01–2.44	0.045
25^th^-50^th^ %ile	1.92	1.21–3.04	0.01	1.43	0.96–2.14	0.068
50-75^th^ %ile	1.59	1.02–2.47	0.04	1.00	0.68–1.46	0.99
75-100^th^ %ile	Reference	Reference	Ref	Reference	Reference	Ref
SWYC^c^ Results:						
-Positive SWYC	4.45	3.21–6.17	<0.001	4.85	3.39–6.92	<0.001
-Negative SWYC	Reference	Reference	Ref	Reference	Reference	Ref
M-CHAT-F^d^ Score (continuous)	1.01	0.98–1.05	0.51	1.10	1.07–1.15	<0.001
						Ref
Age in Months	0.99	0.94–1.03	0.49	1.03	0.99–1.07	0.21

^a^All models adjusted for time from study initiation

^b^Quartile data for income included in models, as continuous data led to poorer model fit given distribution of sample

^c^Survey of Wellbeing in Young Children (SWYC)

^d^Modified CHecklist for Autism in Toddlers (M-CHAT-F)

## Discussion

Universal screening holds the potential to improve care and reduce disparities, but it is insufficient on its own. Despite a high rate of screening [23], we found that strict adherence to guidelines for completing the follow-up interview and immediate referral upon a positive M-CHAT-F could be improved. Patient demographic characteristics were associated with immediate referral for EI and ASD/additional evaluations.

The M-CHAT-F’s follow-up interview reduces the false positive rate [28], and was available to clinicians but not required. Clinicians did not administer the follow-up interview to many children with moderate-risk scores for whom it was indicated. This points to an ongoing challenge with screening implementation with a two-step screener in busy practices. Other research groups have found that even in research settings, administration of the follow-up interview proves challenging [26], and in clinical practice is not regularly being administered at all [19, 20]. In this context, our follow-up interview rate of 42% was higher than for other reported clinical samples.

Completing the follow-up interview was more likely for children with public insurance and lower income, or for children who concurrently screened positive on a general developmental screener. Clinicians may have based their decision to complete the follow-up interview on concerns about added expense to families. Some private insurance companies do not cover ASD screening, and families are billed directly; but public insurance companies do. While it is part of the standard workflow to have parents complete the questionnaire, clinicians may have chosen not to administer the follow-up interview or bill for the screen for privately insured families (who are also more likely to be higher-income). On the other hand, we expected that clinicians would have been more likely to bypass the follow-up interview entirely, opting to refer children who concurrently screened positive on the SWYC, but these children were *more* likely to have the follow-up interview administered. Perhaps clinicians paid increased attention to developmental concerns in children when alerted to a positive SWYC, and used the follow-up interview as a probe to find out more about the child’s developmental status.

Importantly, when administered, most (94%) children given the follow-up interview subsequently screened negative. Other screening studies have found much higher rates of positive follow-up interviews when administered in research settings (25–54%) [25, 26]. Our prior analyses in a similar sample that found that children who received the follow-up interview were significantly less likely to have ASD [23]. Taken together, these findings suggest that the M-CHAT follow-up interview was used in this population when the accuracy of the initial screen was questioned and a false positive was suspected. However, although children who continued to screen positive after the follow-up interview had the highest rates of ASD referrals, no scenario led to 100% adherence to referral recommendations, suggesting there is more to learn about what leads to a referral, and/or more opportunities to standardize care.

In our sample drawn from a large, diverse set of pediatric practices, 26% had already been referred to EI before the positive M-CHAT-F, and non-white and younger children were less likely to access EI early. Because families can self-refer to EI when they have a concern and have to agree to pursue EI when a physician makes a referral, parental beliefs about development and the utility of EI services likely play an important role in the representation of children within the EI system. We were unable to identify whether EI was initiated by a self-referral or as a result of a medical recommendation at a prior visit. However, we know that family beliefs about development and EI may vary by a family’s racial or ethnic background [29], and may have, along with logistic or other barriers, contributed to differences in early EI access in our population.

Another 31% of our sample were newly referred to EI the day of the positive screen. Across the entire sample, only 3.7% of children left the screening visit with all 3 recommended referrals in place (a new or existing EI referral, new audiology referral, and new ASD/additional evaluation referral) [5, 6]. Importantly, although we previously found that accuracy of the M-CHAT-F was lower than previously reported at detecting ASD, the M-CHAT-F performed better at detecting any developmental delay or concern; the positive predictive value of a positive screen for any developmental delay was 72.4% [23], indicating that children with a positive screen should be referred for evaluation given the high likelihood that they have a developmental issue (including possible ASD).

In clinical care, providers have to make judgments about how guidelines may or may not fit the patient scenario in front of them, including logistic and financial considerations for the family. Clinical judgment appeared to play a role in who received the follow-up interview, with an apparent bias toward administering the follow-up interview when a false positive was suspected, which may reflect the best care for that scenario since it is an immediate, low-cost, and evidence-based practice which informs the decision not to refer. Thus, while only 10% of children administered the follow-up interview continued to screen positive, we cannot extrapolate the same proportion would hold true for children not administered the follow-up interview.

Similarly, given that our group found lower accuracy of the M-CHAT-F among non-white children, girls and children from households where languages other than English are spoken [23], non-referral in response to a positive M-CHAT-F in these groups may have ultimately been the provider’s best care option, given the low risk of ASD and potential cost in time and money to the family to obtain an evaluation. Conversely, M-CHAT-F accuracy was also lower among children from lower-income households, and children with public insurance [23], but we found that children with lower income were more likely to be referred for additional evaluation, perhaps because a low-cost medical evaluation, accessible through public insurance and with evidence that it will provide helpful information about the child’s development regardless of an ASD diagnosis or not, was the best care option in this scenario.

Thus, multiple factors must be studied to determine how to best support early screening and identification. If the psychometric properties (e.g. false positive rate, sensitivity, positive predictive value) of the screen differs based on patient characteristics, including differences in how parents report concerns [30] or screen-positive rates [31], non-referral may be appropriate. However, these same groups have traditionally been under-identified with ASD [10], and failure to refer children may contribute to their decreased likelihood for early identification. Furthermore, it remains unclear what factors contribute to a pediatrician’s clinical impression about ASD risk and how these factors drive decision-making about referral.

In some cases, the differences in referral may relate to language barriers or logistical challenges to care. For example, children who lived in a household where only English was spoken were more likely to be referred to EI or for additional ASD evaluation, which may reflect a provider’s perception that screening results are not as accurate for non-English speakers, or that care in English is easier to access than care in other languages.

Other differences did not seem to relate only to logistical barriers, however. Males were more likely than females to be referred to EI or for audiology, but equally likely for ASD evaluation. Given the increased rate of ASD diagnosis among males [17], it does not seem that perceived risk of ASD is the only factor driving referral for ASD evaluation. Furthermore, at least in our data, referral rates for ASD evaluations were similarly low for both girls and boys. If this practice pattern is true in other populations, this could suggest that differences in referral for ASD evaluation–at least in response to toddler screening—are not major contributors to lower diagnosis rates among girls. Black children were less likely to be newly referred to EI, but more likely to be referred to audiology. Asian children were less likely to be referred for additional ASD evaluation, but no other race or ethnicity was significantly associated with referral. This pattern of referrals suggest that decision-making in response to a positive screen is complex and merits additional study.

Disentangling the contribution of race and SES (socio-economic status) to disparities can be difficult. One strength of this current study is its use of family self-reported race as well as two measures of SES: insurance payer and median household income at the census-tract level to determine the individual effects of race and SES on EI referral rates. However, each of these SES measures appeared to function differently with respect to referrals, with privately insured children (generally considered of higher SES) less likely to be referred to EI and those from neighborhoods with lower household income more likely to be referred for ASD evaluation and audiology. This may speak to the increased accessibility of children with higher SES to medical evaluations, but those with public insurance more likely to access publicly available (and free or low-cost) EI services. Parental education also is an important component of SES, but this information is not systematically collected in the EHR. With this limitation, our results still suggest that factors other than SES drive the lower proportion of non-white children referred prior to or on the day of the positive screen.

We are not the first to find low rates of referrals. A recent study found that 31% of children who screened positive were referred for diagnostic evaluation, 20% to EI, and 36% to audiology. In a public primary care setting serving predominately Hispanic patients, approximately 28% of children were referred for additional evaluation after a positive M-CHAT-F [32]. In that sample, children with higher M-CHAT-F score and positive general developmental screen were more likely to be referred, as was seen in our analyses. In a study of 17 sites nationwide, referrals after a positive developmental screen ranged from 48–78% depending on the clinic, which led the authors to conclude that screening and referrals each need their own workflow procedures [33]. CHOP has established relatively automated workflows for screening and EI referrals, but not for audiology or ASD evaluation referrals. These findings may motivate that change to be made. Our findings regarding the low rates of referral to audiology after positive screen may conform to new recommendations [7] about referral for a hearing assessment only if there are concerns about language or hearing.

A number of study limitations should be mentioned. First, CHOP is a large and diverse network, but may not reflect practices in other settings or locations, especially given its status as a teaching hospital. Second, our analyses examined only pre-existing EI referrals and referrals made the day of the screening visit–not what else might have happened at prior or subsequent visits. However, earlier intervention is the goal and assessing immediate actions taken in response to screening remains important. Finally, data obtained from the EHR may not fully reflect the extent to which parental concerns, co-occurring conditions, risk factors, or physician assessment of child’s development confounded the associations with referrals.

It was beyond the scope of this paper to systematically determine whether families followed recommendations, or each child’s diagnostic outcome over time. Thus, we do not know whether these disparities in same-day referrals led to different long-term outcomes. The focus of this study was on physician behavior during the visit, however, and our results do suggest families are having different experiences upon receiving a positive ASD screen. When trying to identify possible explanations for disparities in the average age of ASD diagnosis, our results shed light on one possible step in a multi-step process.

While we identified differences in referrals based on child variables, future work is needed to determine the accuracy of physician’s decisions, as well as other factors, such as parental concern, informal clinical observation, logistical barriers, perceptions of the family’s ability and interest in accessing additional care, or possible unconscious bias that are considered when evaluating a young child’s risk of ASD. As these children age, we may be able to more systematically determine who in this cohort is diagnosed with ASD among those children who continue to receive care at CHOP. However, interpretation of diagnostic outcome will be challenging, as it will be impossible to know if children were diagnosed because they were referred, or if they were referred appropriately because of early observable signs of ASD. Qualitative studies may shed light on the process of pediatrician decision-making about ASD screening and referral. Other authors have made the case that not all children with positive screens need to be referred [34, 35], but if demographic factors are affecting referral decisions, disparities in ASD identification and intervention may perpetuate.

### Conclusions

Within a large primary care network that implemented universal developmental and ASD-specific screening, we found that while administration of the parent questionnaire was high, use of the M-CHAT follow-up interview and rates of immediate referral to EI, audiology, and to ASD/additional evaluation were often low. Most immediate referrals were to EI, but even EI referral rates differed based on sex, family’s language, developmental presentation, SES, and race. The reasons behind the disparities in referral after positive ASD screening are unclear, but these differences may be contributing to disparities in the identification of ASD among girls and non-white children that have been described in the literature. Additional research is needed to understand provider decision-making in response to positive ASD screening, to determine if likelihood of immediate referral improves the equity of diagnosis of developmental delays and/or ASD, and to develop interventions to improve the overall rates and equity of ASD referral for intervention and additional assessment after positive ASD screening.

## Supporting information

S1 Data (XLSX)

S2 Data (DOCX)

## Abbreviations

AAP: American Academy of Pediatrics
ASD: Autism Spectrum Disorder
CDC: Centers for Disease Control and Prevention
CHOP: Children’s Hospital of Philadelphia
EHR: Electronic Health Record
EI: Early Intervention
M-CHAT-F: Modified CHecklist for Autism in Toddlers with the Follow up Interview
SES: Socio-economic status
SWYC: Survey of Wellbeing in Young Children
