# Supplementary material for: Adherence to screening and referral guidelines for autism spectrum disorder in toddlers in pediatric primary care
Source: PLoS One. 2020 May 7;15(5):e0232335. doi: 10.1371/journal.pone.0232335 (PMC7205236; doi:10.1371/journal.pone.0232335)
Supplement: S2 Data — (DOCX) [file pone.0232335.s002.docx]

| Variable | Position | Label | Measurement Level | Missing Values |
| --- | --- | --- | --- | --- |
| id | 1 | deidentified id | Scale |  |
| timestart | 2 | Difference between visit date and 1/1/13 | Scale |  |
| Exclusion_Criteria | 3 | Exclusion Criteria | Nominal |  |
| Year | 4 | <none> | Scale |  |
| Already_in_EI | 5 | Previously referred to EI, or receiving EI prior to screening visit | Nominal |  |
| referEI | 6 | Referred to EI in Visit or Family Refused | Nominal |  |
| refer3way | 7 | Refer to EI--3 way | Nominal |  |
| referASDeval | 8 | Refer for additional evaluation | Nominal |  |
| referaudio | 9 | Refer to audiology | Nominal |  |
| anyref | 10 | Any referral made | Nominal |  |
| all_rec_ref | 11 | All recommended referrals made | Nominal |  |
| agemos | 12 | Child in Age in months at time of screen | Scale |  |
| practiceid | 13 | <none> | Scale |  |
| PROVIDER_CAT | 14 | Provider Type Categorical | Nominal |  |
| female | 15 | Female versus male sex- patient | Nominal |  |
| ethnicity | 16 | Ethnicity Binary | Nominal | 999 |
| race4grp | 17 | Race Categorical | Nominal | 999 |
| private | 18 | Private versus Public insurance | Nominal | 999 |
| englishonly | 19 | English-only language versus multiple/non-English | Nominal | 999 |
| county | 20 | County of Family Residence | Nominal | 999 |
| medhouseinc | 21 | Median Household Income | Scale | 999 |
| Median_Household_inc_Err | 22 | Median Household Income- Margin of Error | Scale | 999 |
| medhouseinc_Cat | 23 | Median Household Income Estimates, Quartiles | Nominal | 999 |
| swyc | 24 | SWYC score (continuous) | Nominal | 999 |
| swycfail | 25 | Results of SWYC at screening visit | Nominal | 999 |
| SUM_MCHAT_ITEMS | 26 | MCHAT Score (Total Positive on Parent Questionnaire) | Nominal | 999 |
| mchatmedrisk | 27 | M-CHAT score by risk category | Nominal | 999 |
| follow_up_int | 28 | Follow-Up Interview Administered | Nominal | 999 |
| follow_up_result_pos | 29 | Follow up interview administered, results positive | Nominal | 999 |

| **Variable Values** | | |
| --- | --- | --- |
| Value | | Label |
| Exclusion_Criteria | 0 | Include |
|  | 1 | Visit delayed > 2 weeks |
|  | 2 | Acute Visit |
| Already_in_EI | 0 | No |
|  | 1 | Yes |
| referEI | 0 | No |
|  | 1 | Yes |
| refer3way | 0 | Not referred |
|  | 1 | Referred prior to visit or already receiving EI |
|  | 2 | Referred during visit |
| referASDeval | 0 | No |
|  | 1 | Yes |
| referaudio | 0 | No |
|  | 1 | Yes |
| anyref | 0 | No |
|  | 1 | Yes |
| all_rec_ref | 0 | No |
|  | 1 | Yes |
| PROVIDER_CAT | 1 | Attending Physician |
|  | 2 | Nurse Practitioner |
|  | 4 | Resident |
|  | 999 | Missing |
| female | 0 | Male |
|  | 1 | Female |
| ethnicity | 0 | Not Hispanic or Latino |
|  | 1 | Hispanic or Latino |
|  | 999^a^ | Missing |
| race4grp | 1 | White |
|  | 2 | Black |
|  | 3 | Asian |
|  | 4 | Other |
| private | 0 | Medicaid |
|  | 1 | Private |
| englishonly | 0 | Non-English or Multiple Languages |
|  | 1 | English only |
| medhouseinc_Cat | 1 | 0-25th percentile |
|  | 2 | 26-50th percentile |
|  | 3 | 51-75th percentile |
|  | 4 | 76-100th percentile |
| swycfail | 0 | Swyc Pass |
|  | 1 | Swyc Fail |
| mchatmedrisk | 0 | high risk |
|  | 1 | medium risk |
| follow_up_int | 0 | No |
|  | 1 | Yes |
| follow_up_result_pos | 0 | No |
|  | 1 | Yes |
|  | | |
